# Factors associated with high-utilization in a safety net setting

**DOI:** 10.1186/s12913-017-2209-0

**Published:** 2017-04-14

**Authors:** Julia Bell, Sara Turbow, Maura George, Mohammed K. Ali

**Affiliations:** 1 Hubert Department of Global Health, Emory University Rollins School of Public Health, 1518 Clifton Road NE, Atlanta, GA 30322 USA; 2 Division of General Medicine and Geriatrics, Department of Medicine, Emory University School of Medicine, 49 Jesse Hill Jr Drive SE, Atlanta, GA 30303 USA

**Keywords:** High-utilizers, Underserved populations, Socioeconomic factors, Disparities, Health behavior

## Abstract

**Background:**

Patients with frequent hospital readmissions, or high-utilizer patients (HUPs), are a major driver of rising healthcare costs in the United States. This group has a significant burden of medical illness, but less is known about whether or how social determinants of health may drive their increased healthcare use and poor health outcomes. Our study aimed to define the population of HUPs at a large, safety-net hospital system, to understand how these patients differ from patients who are not HUPs, and to analyze how their demographic, medical, and social factors contribute to their healthcare use and mortality rates.

**Methods:**

For this case-control study, data were collected via retrospective chart review. We included 247 patients admitted three or more times in a single calendar year between 2011 and 2013 and 247 controls with one or two admissions in a single calendar year matched for age, sex, and year of high-utilization. We used multivariable logistic regression models to understand which demographic, clinical, and social factors were associated with HUP status, and if HUP status was independently associated with mortality.

**Results:**

The factors that contributed significant odds of being a HUP included having Medicaid (OR 3.34, 95% CI 1.50, 7.44) or Medicare (OR 3.39, 95% CI 1.50, 7.67), having a history of recreational drug use (OR 2.44, 95% 1.36, 4.38), and being homeless (OR 3.73, 95% CI 1.69, 8.23) The mortality rate among HUPs was 22.6% compared to 8.9% among controls (*p* < 0.0001).

**Conclusions:**

These data show that social factors are related to high-utilization in this population. Future efforts to understand and improve the health of this population need to incorporate non-clinical patient factors.

**Electronic supplementary material:**

The online version of this article (doi:10.1186/s12913-017-2209-0) contains supplementary material, which is available to authorized users.

## Background

As health care costs rise in the United States (US) [1], efforts to decrease spending while improving quality are a focus for providers, health systems, and governments. Hospital readmission rates are under increased scrutiny as they are a major source of health expenditures: in 2011, there were over 3 million readmissions within 30 days of discharge, which were associated with over $41 billion in healthcare expenses [2]. Overall, health care spending is highly skewed to the top five percent of the general population, who account for half of all healthcare costs [3]. The intersection of increased healthcare spending, efforts to improve quality of care, and a focus on decreasing hospital readmissions has led to heightened interest in a group of patients with significantly more healthcare use than their peers: “high-utilizers.”

Research to date has identified that high-utilizers are a medically and socially complex group [4–7]. High-utilizers have a high burden of chronic disease and mental illness [4, 8], often come from lower socioeconomic groups [9], and have limited access to primary and preventive care [10]. They also have higher rates of adverse social determinants of health, such as disability [11] social isolation [12], and food insecurity [7]. Unfortunately, despite their increased healthcare use, high-utilizers have poorer health outcomes and higher mortality rates than their peers [13, 14].

A major goal of this study was to identify the population of high-utilizers of inpatient medical services at an urban, public, safety-net hospital in the Southeastern US. The population at our hospital has a high prevalence of many of the risk factors for high-utilization mentioned above, yet we still have a distinct cohort of high-utilizers. Because of this, we sought to characterize how our high-utilizers differ from our patients who are not high-utilizers. We then analyzed what demographic, medical, and social factors predict high-utilizer status and if being a high-utilizer is associated with increased mortality.

## Methods

### Population and data sources

Data for this study came from the electronic medical record of Grady Health System (GHS) in Atlanta, Georgia [15, 16]. Cases and controls were identified through a master list of patients seen in the Emergency Department (ED) between 2011 and 2013. This list included a patient’s unique Medical Record Number (MRN) and their disposition from the ED (admitted, discharged, or deceased).

For the purpose of this study, high-utilizer patients (HUPs) were defined as those with three or more admissions to inpatient services in a single calendar year, representing the 95th percentile of most inpatient admissions. Between 2011 and 2013, this group included over 3000 individual patients. A total of 250 cases were randomly selected from the initial cohort. Of these, three patients with end-stage renal disease receiving dialysis at GHS were excluded from the sample, as their admissions were attributable to policies requiring them to receive their dialysis sessions in the inpatient setting. This resulted in a final sample of 247 cases.

Controls were defined as any patient with one or two inpatient admissions in a single calendar year between 2011 and 2013. This control group was selected to allow focus on the differences between patients who require hospitalization but are not high-utilizers, a comparison that would be helpful to both clinicians and policy makers. Control patients were selected in a 1:1 ratio from the original master list of patients, and were matched with cases for age, sex, and year of high use. If multiple control patients for a given year matched a case, a random number generator was used to select the control.

IRB approval was obtained from Emory University and the Grady Hospital Research Oversight Committee, and all analyses and study procedures complied with HIPAA regulations.

### Data collection

Data were collected from the GHS Electronic Medical Record (EMR) (Epic Systems Corporation, Verona, WI) via retrospective chart reviews completed between October 2014 and February 2015. Data abstraction was done in accordance with the study protocol (Additional file 1). Patient information was collected and managed using RedCap electronic data capture tools hosted at Emory University [17]. All data collected was restricted to the year of high-utilization for cases or the matched year for controls unless otherwise noted.

Demographic data included sex, birthdate, race/ethnicity, street address, county of residence, zip code, and whether or not the patient was deceased. Patient age was calculated as of the index admission during the year of interest. For analytical purposes, race was converted to a dichotomous covariate (Black and non-Black) for the logistic regression analysis. Patient income was defined by grouping zip codes and classifying household income using the U.S. Census Bureau’s 2009–2013 American Community Survey 5-Year Estimates [18]. Patients were defined as deceased if their death was documented in the medical chart at any point between 2011 and when chart abstraction occurred, or if they were discharged to home or inpatient hospice and had no further follow-up at GHS. Death outside of these two situations was not captured.

Variables abstracted included medical and social factors; these were selected based on literature review [4, 7–12]. Medical information included all current and past diagnoses, reasons for admission, and number of outpatient medicines prescribed. Medical diagnoses and reasons for admission were abstracted from the history and physical (H&P) and discharge summaries. Medical conditions were recorded individually, but were grouped by organ system for analysis; a full list of both diagnoses and their corresponding disease category can be found in Additional file 2. Chronic disease was defined as any cardiac, neurological, hematological, renal, or endocrine diagnosis. Multiple chronic conditions (MCCs) were defined as having 3+ of the aforementioned chronic diagnoses. The number of outpatient medications was defined as the total count of unique outpatient prescriptions.

Weight and height were abstracted from the first H&P in which they were documented. Body mass index (BMI) was calculated using the formula weight (kg)/height^2^ (m).

Non-clinical factors included social characteristics, “habit” variables, and insurance status and payers. Social characteristics included housing status, employment status, and current or past incarceration. These data were collected from H&Ps, administrative charts, or social worker (SW) notes. Tobacco, alcohol, and drug use were classified as “habit” variables and were collected from the same sources. Current use of tobacco, alcohol, or recreational drugs was defined as self-reported use or documentation of a positive urine drug or blood alcohol test in one of the above sources. History of use was positive if a patient had either current use or a self-reported history of use. Negative use was defined as both explicitly stated denial of use as well as no statement or documentation of use. Substance use was only considered as a non-clinical characteristic, it was not grouped under the “psychiatric” disease category. Patients were considered to be insured if they had insurance coverage at any point during the year of interest; specific payer information was collected from discharge summaries and SW notes.

Data regarding health care use included: the number of ED visits and inpatient admissions, admission and discharge dates, and discharge disposition. The number of ED visits included all ED visits, whether or not they resulted in an admission. Disposition was categorized as home, nursing home, sub-acute rehabilitation (SAR), or hospice (home or inpatient). Discharge summaries provided information regarding admission and discharge dates and disposition.

### Analysis

This study included 247 cases (HUPs) and 247 controls. SAS software (SAS Version 9.2, Cary, NC) was used for all statistical analyses. Descriptive analyses were used to compare cases and controls’ demographic, social, and medical characteristics. Chi-square tests of homogeneity and independent t-tests were used to determine whether differences between cases and controls were statistically significant (*p* < 0.05) for categorical and continuous variables, respectively.

We used multivariable logistic regression models to identify which demographic, social, and medical factors were associated with HUP status. For this analysis, HUP status was treated as the primary outcome of interest, while the demographic, medical, and social variables were the exposures. In the main logistic regression models, age and sex were not included as cases and controls were matched on these variables; however, in sensitivity analyses, age and sex were included to determine if point estimates were affected by their exclusion. Univariate and multivariate regressions were run to determine associations between covariates and high-utilizer status. First, each covariate was entered into preliminary bivariate logistic regression models to determine the association with HUP status. Covariates were then grouped into categories: demographics, insurance payer, social history, habits, and medical history. Four logistic regression models were created that adjusted for each thematic group sequentially and then for all five covariate groups together. Alpha was set at 0.05. Odds ratios and confidence limits were used as measures of association, and r-squared values were used to determine the variance explained by each model.

A parsimonious model was created using forward stepwise selection with the fully adjusted logistic regression model. The significance level for entry into the model was set at 0.10 [19]. The Hosmer-Lemeshow Goodness of Fit Test was used to evaluate the model’s fit, and t-squared values were used to determine the total variance explained by the parsimonious model.

To determine how clinical diagnoses affected associations between covariates and HUP status we created fully adjusted logistic regression models that varied by medical history; this allowed us to compare models that included all diagnoses, any chronic diagnosis, or the diagnosis of multiple chronic conditions. In this paper we discuss the multivariate model factoring in multiple chronic conditions; the two additional models are provided in Additional file 3.

We conducted an additional sensitivity analysis that excluded deceased patients to determine whether removing those patients where death might have been imminent, a proxy for illness severity, would affect the associations between exposures and HUP status.

To investigate if HUP status, independent of other demographic, social, and medical factors, was associated with higher odds of mortality, we pooled cases and controls, and HUP status was treated as the primary exposure for mortality. For this analysis, we performed an additional logistic regression model with the same methods described above. Age and sex were included in these models.

## Results

Demographic and social characteristics are presented in Table 1. HUPs were more likely to have Medicaid as their insurance (51.1% vs. 28.2%, *p* < 0.0001), and to come from the lowest income quartile. More than 33% of all HUPs were in the lowest income quartile, compared to only 22% of controls (*p* = 0.002). Compared to control participants, HUPs were also more likely to be homeless (19% vs. 4.5%; *p* < 0.0001), and use alcohol, tobacco, or recreational drugs.

**Table 1:** Patient social and demographic characteristics

	Cases (*n* = 247)	Controls (*n* = 247)
Characteristic	%	%
Demographic information		
Age, years mean (std. dev.)	55.28 (15.72)	56.09 (15.62)
Sex		
Male	56.68	56.68
Female	43.32	43.32
Race		
Black	88.26	76.52^a^
White	7.69	18.22
Hispanic	2.02	4.86
Asian	0.81	0
Other	0.81	0.4
Not specified	0.4	0
Community-level median household income, dollars		
0–27,651	33.74	21.72^a^
27, 652–39,421	28.05	23.77
39, 422–48,093	19.11	23.77
48,094–139, 543	19.11	30.74
Insurance information		
Insurance status		
Uninsured	27.13	36.84^a^
Insured	72.87	63.16
Payers		
Medicaid	51.11	28.21^a^
Medicare	34.44	20.51
Medicare and Medicaid	10	18.59
Private/Other	3.89	28.21
Social history		
Alcohol use		
Current use	43.72	37.65^a^
History of use	52.63	42.11^a^
Alcohol-related admission	38.89	33.33
Tobacco use		
Current use	42.11	34.41^a^
History of use	68.42	46.56^a^
Substance use		
Current use	28.34	9.72^a^
History of use	36.44	12.55^a^
Homeless	19.03	4.45^a^
Employed		
No	70.85	38.21^a^
Yes	3.64	9.35
Incarceration		
Currently incarcerated	6.48	3.24
History of incarceration	12.15	5.26^a^

^a^significant at alpha = 0.05

The clinical profile of HUPs was also quite different from control participants (Table 2). High-utilizer patients had a higher prevalence of diseases in eleven out of thirteen disease categories, including cardiac, pulmonary, infectious, and psychiatric diseases. There was no statistically significant difference in the prevalence of trauma or musculoskeletal diagnoses (*p* = 0.15 and *p* = 0.08, respectively) between cases and controls.

**Table 2:** Patient diagnoses by disease category and organ system

	Cases (*n* = 247)	Controls (*n* = 247)
Diagnosis	%	%
Chronic disease	97.58	76.11^a^
Multiple chronic conditions	52.22	17.81^a^
Neurological	46.15	25.51^a^
Cardiac	82.59	61.54^a^
Pulmonary	46.96	19.03^a^
Gastro-intestinal	49.39	19.84^a^
Hematological	61.13	17.00^a^
Infectious disease	25.91	8.50^a^
Renal	39.68	13.36^a^
Gynecological/Urological	20.65	12.96^a^
Musculoskeletal	28.34	21.46
Psychiatric	36.44	18.22^a^
Endocrine	44.53	27.13^a^
Trauma	3.64	6.48
Rheumatology/Nutritional Deficiencies/Other	12.55	11.74
Weight status		
BMI, mean (std. dev.)	27.06 (8.04)	27.76 (7.11)

^a^significant at alpha = 0.05

There were significant differences in other health care services utilization between HUPs and controls (Table 3). HUPs had a higher number of ED visits (mean of 8.1 vs. 1.7, *p* < 0.0001) and outpatient medications (mean of 18.4 vs. 6.3, *p* < 0.0001). High utilizer patients were also more likely to be discharged to SAR (11.3% vs. 6.1%, *p* = 0.04) and nursing homes (22.3% vs. 5.3%, *p* < 0.0001). Interestingly, neither length of stay (5.9 vs. 5.6 days; *p* = 0.47) nor time to readmission (46.5 vs. 59.9 days; *p* = 0.06) were statistically different between HUPs and controls.

**Table 3:** Health care utilization (ED visits, inpatient admissions, outpatient medications, length of stay, time to readmission, disposition) and mortality in cases and controls

	Cases (*n* = 247)		Controls (*n* = 247)	
Outcome	Mean (Std.Dev.)	Cumulative	Mean (Std.Dev.)	Cumulative
ED visits, year of high use				
All years	8.11 (7.36)	2003	1.74 (1.58)	431^a^
2011	8.31 (7.01)	789	1.62 (1.21)	154^a^
2012	6.88 (5.33)	585	1.89 (2.01)	164^a^
2013	9.39 (9.61)	629	1.74 (1.42)	113^a^
Number of admissions, year of high use				
All years	4.55 (2.01)	1123	1.23 (0.42)	305^a^
2011	4.79 (2.11)	321	1.26 (0.44)	82^a^
2012	4.33 (1.88)	368	1.24 (0.43)	108^a^
2013	4.57 (2.05)	434	1.21 (0.41)	115^a^
Outpatient medications, year of high use				
All years	18.39 (7.50)	4543	6.28 (4.97)	1550^a^
2011	17.99 (7.36)	1709	5.97 (4.98)	567^a^
2012	18.26 (7.05)	1552	6.46 (4.51)	562^a^
2013	19.13 (8.26)	1282	6.48 (5.55)	421^a^
	Mean (Std.Dev.)	Median	Mean (Std.Dev.)	Median
Length of stay (days)	5.90 (6.99)	4	5.57 (6.99)	4
Time to readmission (days)	46.5 (53.4)	26	59.87 (66.22)	30
	%		%	
Disposition				
Nursing home	22.27		5.26^a^	
Sub-acute rebab	11.34		6.10^a^	
Hospice	9.31		2.83^a^	
Deceased				
No	77.33		91.09	
Yes	22.67		8.91^a^	

^a^significant at alpha = 0.05

In logistic regression models examining which factors contributed to high-utilizer patient status (Table 4), non-medical factors that contributed significant odds of being a HUP included being insured by Medicaid (OR 3.34, 95% CI 1.50, 7.44) or Medicare (OR 3.39, 95% CI 1.50, 7.67), having a history of recreational drug use (OR 2.44, 95% 1.36, 4.38), and being homeless (OR 3.73, 95% CI 1.69, 8.23). All of these associations were preserved when age and sex was included in the sensitivity analysis. Multiple chronic conditions also factored into higher odds of HUP status, with MCCs contributing between to almost five times higher odds (4.75, 95% CI 2.96, 7.63) of being a HUP.

**Table 4:** Regression analysis demonstrating demographic, social, and medical factors associated with high utilizer patient status and mortality

	HUP status Group A^a^	HUP status Group B^b^	Mortality
	OR (95% CI)	OR (95% CI)	OR (95% CI)
HUP status	N/A	N/A	3.27 (1.74, 6.13)
Age (Years)	N/A	N/A	1.05 (1.02, 1.07)
Sex	N/A	N/A	
Male			0.94 (0.53, 1.67)
Female			1
Race			
Non-Black	1	1	1
Black	1.45 (0.79, 2.66)	2.03 (1.00, 4.11)	0.68 (0.32, 1.45)
Community income level, dollars			
0–27,651	1.49 (0.81, 2.73)	1.37 (0.71, 2.65)	1.40 (0.61, 3.22)
27,652–39,421	1.18 (0.63, 2.18)	1.11 (0.57, 2.18)	1.95 (0.84, 4.52)
39, 422–48,093	1.03 (0.54, 1.96)	0.73 (0.35, 1.51)	2.24 (0.95, 5.26)
48, 094–139,543	1	1	1
Payer			
Medicaid	3.34 (1.50, 7.44)	3.07 (1.29, 7.30)	1.79 (0.61, 5.19)
Medicare	3.39 (1.50, 7.67)	2.87 (1.18, 7.00)	1.16 (0.42, 3.23)
Medicare and Medicaid	1	1	1
Private	0.46 (0.16, 1.31)	0.39 (0.12, 1.25)	1.07 (0.28, 4.08)
Self/None	1.53 (0.71, 3.31)	1.32 (0.57, 3.04)	1.83 (0.63, 5.37)
History of alcohol use			
Yes	0.79 (0.48, 1.29)	0.84 (0.49, 1.44)	2.05 (1.07, 3.93)
No	1	1	1
History of tobacco use			
Yes	1.45 (0.90, 2.33)	1.28 (0.75, 2.17)	1.12 (0.62, 2.01)
No	1	1	1
History of substance use			
Yes	2.44 (1.36, 4.38)	2.55 (1.37, 4.75)	0.56 (0.27, 1.18)
No	1	1	1
Homelessness			
Yes	3.73 (1.69, 8.23)	3.84 (1.67, 8.82)	0.68 (0.26, 1.75)
No	1	1	1
History of incarceration			
Yes	1.38 (0.59, 3.27)	1.42 (0.59, 3.41)	0.47 (0.13, 1.78)
No	1	1	1
Medical history			
Multiple chronic conditions	4.75 (2.96, 7.63)	4.64 (2.73, 7.88)	1.38 (0.78, 2.44)

^a^Group A includes all HUPs

^b^Group B removes deceased HUPs

Non-medical factors (demographic, payer, and social history exposures) contributed to 21.5% of the explained variance of HUP status; multiple chronic conditions contributed an additional 7%. In sum, the medical and non-medical exposures in our analysis explained 28.5% of the variance of high use (Table 5).

**Table 5:** Comparison of explained variance for three regression analyses exploring demographic, social, and medical factors associated with high utilizer patient status and mortality

Model	Variables	R square	Added variance
Adjusted multivariate, all disease categories			
A	Demographic	0.042	__
B	Demographic, Payer	0.146	0.104
C	Demographic, Payer, Social Characteristics	0.185	0.039
D	Demographic, Payer, Social Characteristics, Habits	0.215	0.03
E	Demographic, Payer, Social Characteristics, Habits, All Diagnoses	0.443	0.228
Adjusted multivariate model, chronic disease			
A	Demographic	0.042	__
B	Demographic, Payer	0.146	0.104
C	Demographic, Payer, Social Characteristics	0.185	0.039
D	Demographic, Payer, Social Characteristics, Habits	0.215	0.03
E	Demographic, Payer, Social Characteristics, Habits, Chronic Diagnosis	0.257	0.042
Adjusted multivariate model, multiple chronic conditions			
A	Demographic	0.042	__
B	Demographic, Payer	0.146	0.104
C	Demographic, Payer, Social Characteristics	0.185	0.039
D	Demographic, Payer, Social Characteristics, Habits	0.215	0.03
E	Demographic, Payer, Social Characteristics, Habits, Multiple chronic conditions diagnosis	0.285	0.07

Demographic variables: Race, Income

Payer variables: Medicaid, Medicare, Medicaid/Medicare, Private Insurance, Self/None

Social variables: Homelessness, Incarceration

Habit variables: Alcohol use, substance use, tobacco use

In analyses of the relationship between HUP status and mortality (Table 4), being a high-utilizer was associated with three times higher odds of mortality (OR 2.99, 95% CI 1.77, 5.09). Adjusted for multiple chronic conditions, the association between HUP status strengthened to more than three times the odds of mortality compared to non-HUP patients (OR 3.27, 95% CI 1.74, 6.13).

## Discussion

In this study, we identified the key demographic, medical, and social factors that differentiate the population of high-utilizers of inpatient services at our urban, safety-net hospital from other patients who require less hospitalizations but may not face the same social obstacles. We noted that high-utilizer patients exhibited increased use of the emergency department, a sign that they may not have good access to primary and preventive care. High-utilization itself was associated with 2–3 times higher risk of mortality.

We found that high-utilizers have an increased burden of chronic disease and mental health diagnoses, come from lower income quartiles, and have higher rates of negative social determinants of health including alcohol, tobacco, and drug use, homelessness, and unemployment. We noted that non-medical factors, especially payer source (Medicaid, Medicare, or self-pay), history of substance use, and homelessness were most strongly associated with HUP status. Furthermore, non-medical factors accounted for a nearly triple proportion of the variance in HUP status as multiple chronic conditions.

These findings support other research exploring how social factors impact health care use, giving further evidence to the argument that interventions to reduce frequent inpatient admissions need to focus on social determinants of health. Patients coming from low-income neighborhoods are admitted to hospitals at a higher rate than their higher-income counterparts and have longer lengths-of-stay, indicating the impact community-level factors can have on health service utilization [20]. Homeless patients have a higher prevalence of both acute and chronic conditions, and are more likely to be frequently admitted to inpatient services [21]. These social and behavioral barriers show that for utilization to decrease, interventions should target not only the primary care level to prevent exacerbation of chronic conditions, but also non-clinical obstacles to good health and appropriate use of services.

The importance of homelessness and history of substance use in this study mirror the social obstacles described in high-utilizer literature from the Center for Medicare and Medicaid Services (CMS), the Camden Coalition, and the Robert Wood Johnson Foundation [10, 22, 23]. Our research further strengthens the concept that the highest-cost patients often face manageable medical conditions made difficult in the context of complex social barriers.

Despite their increased healthcare use high-utilizers have poorer health outcomes, as evidenced in our analysis by their significantly higher mortality rate. Substantially higher rates of mortality in high-utilizer patients have not been extensively studied; the relationships between hospitalizations and mortality is complex. Over 50% of HUPs in our sample had multiple chronic conditions (MCCs), and differences in mortality between patients with and without MCCs were significant. Data from 2009 found that adults with more than three chronic conditions who were discharged from inpatient care had a mortality rate of 3.1%, while adults discharged with zero or one chronic disease had a mortality rate of 1.9% [24]. Our mortality rates were much higher, potentially because of the additional social barriers our high-utilizers experienced. Given the multitude of factors that impact mortality, more research is needed focusing specifically on risk factors for mortality in high-utilizers and interventions or policies that can address preventable deaths.

While the insights into high health care utilization gained in this study are promising, creating a truly comprehensive model of risk factors that predict high utilizer status demands further investigation. On a federal scale, CMS addressed the need to better understand high utilizers through a five-year, $157 M Accountable Health Communities model that aims to systematically identify social factors that impact health care costs and utilization in public insurance beneficiaries [25]. Qualitative studies to identify non-medical risk factors that cannot be assessed through chart review, such as health literacy, educational attainment, and social support will be a key part of improving our understanding of drivers of high-utilization.

This study had some limitations that merit discussion. First, the use of a retrospective chart review is inherently limiting. Researchers have no control over how the data were collected from patients during the clinical encounter, nor how the data were first entered into the EMR. For example, we were not able to consistently capture disease severity (e.g. cancer stages), which prevented us from understanding how disease severity impacts utilization on a more granular level. The research was also limited to one health system, and may not have captured HUPs who are admitted to multiple hospitals in a given year, or may have included patients as controls who were actually HUPs who primarily sought care at other hospitals. Similarly, patients who were not admitted through the ED may have been missed. In addition, there may be misclassification of exposures where no response is provided or documented (e.g., for substance use or other sensitive variables) and this may lead to lower prevalence estimates of some exposures. Insufficient power in the regression analyses may have contributed to weak associations with income and incarceration that were significantly different in cases and controls, but were not retained in the final model. Indeed, given the whole population accessing care at this safety net hospital is of generally low socioeconomic status, this may influence the magnitude of associations between income and high use. Finally, our model only explained 28.5% of the variance of high-use. This is likely due to patient- and health-system factors that cannot be gleaned from chart review and supports the need for additional research, including qualitative studies, to explore these other variables.

## Conclusions

To our knowledge, this research is the only case-control study in a US safety-net health system that focuses on the demographic, social, and medical contributors to high utilization of inpatient health services. High-utilizer patients are arguably among the most marginalized members of society, consistently facing challenges that compromise their physical health and overall well-being. Our results show that utilization is not exclusively influenced by medical conditions, but instead is also impacted by social barriers to good health such as homelessness and substance use. The information in this study can help advance policy, programs, and further research exploring the root causes of high utilization of inpatient services. Hopefully, it will also become part of a greater body of evidence that drives health care reform efforts targeted at eliminating social and structural disparities that result in disproportionately poor health outcomes for marginalized members of society.

## Additional files

Additional file 1: Chart Abstraction Protocol. Includes detailed instructions on how and where to find each data element in the patient’s electronic medical record. (DOCX 130 kb)

Additional file 2: Classification of patient diagnoses by organ system/disease category. Table including all medical diagnoses with associated organ system/disease category. (DOCX 120 kb)

Additional file 3: Multivariate regression analyses with individual diagnoses and chronic disease variables. Regression tables of multivariate analyses factoring in all diagnoses and analyses factoring in chronic disease. (DOCX 23 kb)

## Abbreviations

CMS: Center for Medicare and Medicaid Services
ED: Emergency Department
EMR: Electronic medical record
GHS: Grady Health System
H&P: History & Physical
HUP: High-utilizer patient
MCCs: Multiple chronic conditions
MRN: Medical Record Number
SAR: Sub-acute rehabilitation
SW: Social worker
US: United States
