# Supplementary material for: Factors associated with high-utilization in a safety net setting
Source: BMC Health Serv Res. 2017 Apr 14;17:273. doi: 10.1186/s12913-017-2209-0 (PMC5391601; doi:10.1186/s12913-017-2209-0)
Supplement: Supplementary file 1 — Chart Abstraction Protocol. Includes detailed instructions on how and where to find each data element in the patient’s electronic medical record. (DOCX 130 kb) [file 12913_2017_2209_MOESM1_ESM.docx]

Chart Abstraction Protocol

- Log into an outpatient environment in Epic (ex. Green/Purple/Orange Pod).
- Open the patient’s record in RedCap.
- Each time you complete a form for a patient, make sure to mark it as “complete” in the drop-down box at the bottom, and don’t forget to save it!
- Please use all lowercase letters for free text whenever possible.

1. Using the chart review button, type the MRN into the “patient lookup” box.
2. Fill in the Demographics form from information on the “patient snapshot” page.
   1. County and Race/ethnicity can be found by clicking on where it says “Demographics” above the patient’s name and then clicking on “Clinical Information.”
   2. If the patient is deceased, the date of death will be written in the same box as their name on the snapshot screen.
3. On the “Medical and Social Data” form, check the box for the year of high-utilization that you are analyzing.
4. In Epic, click on chart review 🡪 notes tab
5. Apply a filter by clicking filters 🡪 write in the dates that you want to look at (ex. 1/1/12 to 12/31/12) 🡪 click “category” 🡪 H&P
   1. You can save this filter to make it easier to apply later.
6. Sort by encounter date.
7. Fill in the number of admissions field based on the number of H&Ps.
8. Look through the H&Ps and fill in the diagnosis fields, as well as the checkboxes for mental health diagnoses and medical diagnoses.
   1. This is also the best place to find information about substance use, homelessness, employment, height, and weight, home O2, chronic Foley, etc.
   2. If the patient has a diagnosis that is one of the checkboxes, you do not need to repeat it in the free text boxes.
9. For the rest of the social and medical data, you should have it from the H&Ps, although you may need to look at discharge summaries as well to fill in the blanks.
10. Click on the “medications” tab and apply your date filter. Uncheck the box that says “current meds only.” Click “generic drug name.” Click on each medicine and see if there are any “AMB” prescriptions. Record the number of unique “AMB” prescriptions within the year.
    1. Supplies do not count (i.e. insulin syringes, lancets, etc.)
    2. Different formulations of the same medicine count, ex. Lantus and aspart would count as two medications.
11. Click on the imaging tab and apply the date filter you used above.
12. Click study status 🡪 final 🡪 sort by name
13. Fill in the type and number of imaging studies that the patient had during the year you are analyzing.
    1. Please note that IR procedures will have multiple procedures listed for the same date, please only count each date once.
    2. TTE/TEE count as ultrasounds.
    3. Heart catheterizations may be listed here but they should be recorded under surgeries.
14. Click the “surgeries” tab to see if the patient had any surgeries.
    1. Look in the notes tab as well under “procedures” and for any “op notes” for any other surgeries that may not be recorded elsewhere.
    2. Later, when you are reviewing the discharge summaries, keep your eyes peeled for any procedures that may be documented in the d/c summary but not elsewhere.
15. Look at the social work notes for documentation of insurance status.
16. For PCP, click on “encounters” and apply your date filter.
    1. Look under department specialty for internal medicine, family medicine, infectious disease (if they go to Ponce clinic), and geriatrics.
    2. Count the number of appointments or office visits (do NOT count encounters marked “orders only” or “telephone” or where an appointment was canceled because of the provider) and record both the total visits and the number of missed appointments.
    3. If they have never been to a PCP appointment, look to see if they have ever been seen as an outpatient.
    4. At this point, I also look to see if there are any outpatient mental health notes and if so, how many visits the patient had.
17. For number of ED visits, go back to the notes tab and uncheck H&P. Scroll down and click “ED provider notes.” There is at least one ED provider note per ED visit (if the patient was actually seen), so you can just count the number of separate visits under this screen.
18. For mental health referral, there are three places to look for this:
    1. Look under the “referrals” tab
    2. Look and see if they have seen a mental health provider as an outpatient during the year you are analyzing.
    3. Look and see if a mental health referral is documented in a discharge summary.
19. Fill in other data like chronic Foley, trach, and discharge to NH/SAR/Hospice as appropriate.
20. From each discharge summary, record the date of admission, date of discharge, if the patient left AMA or not, their chief complaint, reason for admission, and what service they were admitted to (medicine, ICU, surgery, psych, etc.).
    1. Make sure the number of discharge summaries correlates with the number of admissions your listed on the previous form.
21. Click complete and save form.
