# Supplementary material for: Factors associated with high-utilization in a safety net setting
Source: BMC Health Serv Res. 2017 Apr 14;17:273. doi: 10.1186/s12913-017-2209-0 (PMC5391601; doi:10.1186/s12913-017-2209-0)
Supplement: Supplementary file 2 — Classification of patient diagnoses by organ system/disease category. Table including all medical diagnoses with associated organ system/disease category. (DOCX 120 kb) [file 12913_2017_2209_MOESM2_ESM.docx]

Classification of patient diagnoses by organ system/disease category.

| **Organ System/Disease Category** | **Diagnosis** |
| --- | --- |
| Neurological | Autism, Bell's palsy, blindness, benign paroxysmal positional vertigo, brain mass, carpal tunnel, cerebral aneurysm, cervical myelopathy, cervical spinal stenosis, cervical spine fracture, CNS vasculitis, cognitive impairment, craniotomy  cerebrovascular accident, deafness, dementia, developmental delay, empty sella syndrome, frontal lobe dementia, hydrocephalus, intracerebral hemorrhage, intracranial hemorrhage, memory loss, meningioma, migraines, multiple sclerosis, neurofibroma, neuropathy, normal pressure hydrocephalus, paraplegia, Parkinson’s disease, peripheral neuropathy, post-herpetic neuralgia, pseudoseizures, quadreparesis, restless leg syndrome, sciatica, seizures, spastic quadriplegia, subarachnoid hemorrhage, subdural hematoma, transient ischemic accident, traumatic brain injury, trigeminal neuralgia, vascular dementia, vertigo |
| Cardiology | Abdominal aortic aneurysm, alcoholic cardiomyopathy, afib with RVR, aicd, aicd placement, angina, aortic aneurysm, aortic dissection, aortic insufficiency, aortic valve replacement, atrial fibrillation, atrial septal defect, atrial tachycardia, atrioventricular dissociation, bradycardia, coronary artery bypass graft, coronary artery disease, cardiac arrest, cardiac cirrhosis, carotid artery occlusion, carotid atherosclerosis, carotid stenosis, chest pain, congestive heart failure, cor pulmonale, hypertensive emergency, hyperlipidemia, hypertension, hypertrophic cardiomyopathy, ischemic cardiomyopathy  LAD, left bundle branch block, left ventricular thrombus, left ventricular hypertrophy, myocardial infarction, mitral valve prolapse, mitral valve replacement, mural thrombus, non-ST elevation MI, orthostatic hypotension, pacemaker, peripheral artery disease, PEA arrest, pericardial effusion, pericarditis, peripheral vascular disease, postural orthostatic tachycardia syndrome, sick sinus syndrome, supraventricular tachycardia, syncope, valvular heart disease, venous insufficiency, venous stasis |
| Pulmonary | Asthma, bronchitis, bronchogenic carcinoma, chronic respiratory failure, chronic obstructive pulmonary disease, empyema, hemoptysis, hem thorax, interstitial lung disease, lung cancer, lung mass, lung metastases, malignant pleural effusion, obesity hypoventilation syndrome, obstructive sleep apnea, pulmonary arterial hypertension, periapical abscess, pleural effusion pneumonia, pneumothorax, pulmonary emboli, pulmonary hypertension, pulmonary nodules, restrictive lung disease, sarcoidosis, tracheal stenosis, tracheocutaneous fistula, tuberculosis |
| Gastrointestinal | Abdominal abscess, achalasia, alcoholic hepatitis, antral gastritis, appendicitis, ascites, atrophic gastritis, arteriovenous malformation, clostridium difficile infection, cecal perforation, cholecystectomy, chronic diarrhea, chronic pancreatitis, cirrhosis, colectomy, colon cancer, colon polyp, colon resection, colostomy, colovesicular fistula, common bile duct obstruction, constipation, Crohn's disease, diarrhea, distal pancreatectomy, diverticulitis, diverticulosis, duodenal ulcer, duodenitis, dysphagia, end ileostomy, erosive esophagitis, end-stage liver disease, esophageal dysmotility, esophageal stricture, esophageal ulcer, esophageal varices, esophagitis, gasroparesis, gastric adenocarcinoma, gastritis, gastroenteritis, gastroesophageal reflux disease, GI bleed, H. pylori, Hepatitis B virus, Hepatitis C virus, hematemesis, hemoperitoneum, hemorrhoids, hepatic encephalopathy, hepatic steatosis, hepatocellular carcinoma  hernia, ileostomy, inguinal hernia, intestinal obstruction, lower GI bleed, nonalcoholic, steatohepatitis, ostomy, pancreatitis, partial hepatectomy, peritonitis, portal hypertension, peptic ulcer disease, rectovaginal fistula, retroperitoneal abscess, spontaneous bacterial peritonitis, sigmoid adenocarcinoma, small bowel obstruction, upper GI bleed, variceal bleed, varices, ventral hernia, ventral hernia repair, gastroparesis |
| Hematological | Acute lymphocytic leukemia, acute myelogenous leukemia, anemia, autoimmune hemolytic anemia, basal cell carcinoma, breast cancer, breast mass, coagulopathy  deep venous thrombosis, diffuse large b-cell lymphoma, fibrous histiocytoma, glioblastoma multiforme, cancer of unknown primary, heparin-induced thrombocytopenia, idiopathic thrombocytopenic purpura, laryngeal carcinoma, lymphoma, metastatic breast cancer, monoclonal gammopathy of undetermined significance, monoclonal paraproteinemia  multiple myeloma, myelodysplastic syndrome, myelofibrosis, nasopharyngeal cancer, neutropenia, osteosarcoma, pancytopenia, polycythemia, Protein S deficiency, sickle cell anemia, sickle cell trait, splenectomy, splenic infarct, squamous cell carcinoma of the tongue, thrombocytopenia, thrombocytosis, thromboembolic disease, tonsilar squamous cell carcinoma, unknown GU cancer |
| Infectious Disease | HIV/AIDS, abscesses, candidiasis, cellulitis, chronic osteomyelitis, cytomegalovirus retinitis, cns toxoplasmosis, cryptococcal meningitis, dental abscess, diabetic foot ulcer, disseminated mycobacterium avium infection, dry gangrene , endocarditis, g6pd deficiency, gangrene of finger, gluteal abscess, gonnococcal bacterial endocarditis, herpes zoster meningitis, hidradenitis suppurativa, HIV retinopathy, HIV-associated nephropathy, HSV laryngitis, Kaposi's sarcoma, latent tuberculosis infection, Ludwig's angina, lumbar spinal stenosis, lymphadenopathy, m. kansasii infection, mycobacterium avium, meningitis  MRSA bacteremia, MRSA pneumonia, neurosyphilis, nocardia pneumonia, osteomyelitis, PCP pneumonia, perianal abscess, periorbital abscess, positive PPD, postoperative infection, progressive multifocal leukoencephalopathy, recurrent severe sepsis, recurrent UTI, rheumatic fever, sacral abscesses, scabies, septic arthrtitis, shingles, sinusitis, thrush, UTI, vertebral osteomyelitis, wound infection |
| Renal | acute interstitial nephritis, chronic hydroureter, chronic kidney disease, congenital solitary kidney, contrast-induced nephropathy, end-stage renal disease, hydronephrosis, hyperphosphatemia, hypokalemia, hyponatremia, nephrectomy, nephrolithiasis, nephrostomy tube, nephrotic syndrome, pyelonephritis, renal artery stent, renal cell carcinoma, renal tubular acidosis type 4, staghorn calculi |
| Gynecology/Urology | BPH, cervical cancer, chlamydia, dysmenorrhea, emphysematous cystitis, endometrial cancer, erectile dysfunction, fibroids, genital herpes, gonorrhea, hypospadias, incontinence, menorrhagia, metastatic vaginal, neurogenic bladder, obstructive uropathy, ovarian cancer, ovarian cyst, ovarian torsion, pelvic floor insufficiency, pelvic inflammatory disease, dysfunctional uterine bleeding, preeclampsia, priapism, prostate cancer, syphilis, testicular cancer, ureteral stent, uterine cancer, uterine fibroids, vaginal cancer, vaginitis |
| Musculoskeletal | ankle fracture, ankle pain, arthritis, avascular necrosis, back pain, c3-c6 laminectomy, cervical stenosis, chronic cauda equine, chronic leg pain, chronic pain, chronic wound, clavicle fracture, degenerative disc disease, degenerative joint disease, decubitus ulcer, elbow fracture, elevated CPK, facial fracture, femoral fracture, fibromyalgia, gout, hip fracture, hip replacement, laminectomy, leg amputation, leg fracture, leg ulcers, lumbar spine fracture, mandible fracture, multiple fractures, myopathy, neck hematoma neck pain, neuropathic pain, osteoarthritis, osteopenia, osteoporosis, paresthesias, pathologic fracture, rotator cuff tear, spinal cord compression, spinal stenosis, t12 fracture, tibial fracture |
| Psychiatric | Bipolar disorder, anxiety, depression, drug overdose, alcohol abuse, hypomania, opioid dependence, personality disorder, psychosis, PTSD, schizoaffective disorder, schizophrenia, suicidal ideation, suicide attempt |
| Endocrine | Diabetes mellitus, adrenal insufficiency, diabetic neuropathy, diabetic ketoacidosis, gestational diabetes, hyperglycemic hyperosmolar state, hyperparathyroisim, hyperthyroidism, hypothyroidism, obesity, panhypopituitarism, primary hyperaldosteronism, thyroid nodule, thyroidectomy, toxic multinodular goiter |
| Trauma | Motor vehicle collision, burn injury, compartment syndrome, electrocution, gunshot wound, sexual assault victim, head trauma, stab wound |
| Rheumatology/Nutritional/Other | Allergic rhinitis, angioedema, axillary swelling, B12 deficiency, blindness, cataracts, chiari malformation, domestic abuse, eczema, eye enucleation, failure to thrive, fistula, glaucoma, hearing impairment, hypercalcemia, hypoalbuminemia, IgG-4 deficiency, insomnia, systemic lupus erythematosus, malnutrition, polymyositis, presbycusis, primary polydipsia, rheumatoid arthritis, scleroderma, seasonal allergies, serotonin syndrome, stem cell transplant, vitamin D deficiency, vocal cord dysfunction |
