# Supplementary material for: Factors associated with high-utilization in a safety net setting
Source: BMC Health Serv Res. 2017 Apr 14;17:273. doi: 10.1186/s12913-017-2209-0 (PMC5391601; doi:10.1186/s12913-017-2209-0)
Supplement: Supplementary file 3 — Multivariate regression analyses with individual diagnoses and chronic disease variables. Regression tables of multivariate analyses factoring in all diagnoses and analyses factoring in chronic disease. (DOCX 23 kb) [file 12913_2017_2209_MOESM3_ESM.docx]

| Table S1. Regression analysis demonstrating demographic, social, and chronic disease status associated with high utilizer patient status and mortality | | | |
| --- | --- | --- | --- |
|  | **HUP Status Group A** | **HUP Status Group B** | **Mortality** |
|  | OR (95% CI) | OR (95% CI) | OR (95% CI) |
| **HUP Status** | N/A | N/A | 3.16 (1.71, 5.83) |
| **Age (Years)** | N/A | N/A | 1.047 (1.024, 1.07) |
| **Sex** | N/A | N/A |  |
| Male |  |  | 0.98 (0.55, 1.73) |
| Female |  |  | 1 |
| **Race** |  |  |  |
| Non-Black | 1 | 1 | 1 |
| Black | 1.56 (0.85, 2.87) | 2.21 (1.09,4.46) | 0.65 (0.30, 1.39) |
| **Community Income Level, dollars** |  |  |  |
| 0 - 27,651 | 1.93 (1.07, 3.48) | 1.90 (1.00, 3.59) | 1.44 (0.62, 3.32) |
| 27,652 - 39,421 | 1.32 (0.72, 2.41) | 1.25 (0.65, 2.41) | 1.99 (0.85, 4.63) |
| 39, 422 - 48,093 | 1.22 (0.65, 2.31) | 0.84 (0.41, 1.73) | 2.29 (0.97, 5.41) |
| 48, 094 - 139,543 | 1 | 1 | 1 |
| **Payer** |  |  |  |
| Medicaid | 2.38 (1.11, 5.10) | 2.18 (0.95, 5.01) | 1.71 (0.59, 4.95) |
| Medicare | 3.05 (1.40, 6.66) | 2.65 (1.13, 6.22) | 1.15 (0.41, 3.20) |
| Medicare and Medicaid | 1 | 1 | 1 |
| Private | 0.50 (0.18, 1.40) | 0.44 (0.14, 1.38) | 1.21 (0.31, 4.69) |
| Self/None | 1.30 (0.62, 2.72) | 1.15 (0.51, 2.58) | 1.84 (0.63, 5.41) |
| **History of Alcohol Use** |  |  |  |
| Yes | 0.78 (0.49, 1.26) | 0.86 (0.51, 1.46) | 2.03 (1.06, 3.89) |
| No | 1 | 1 | 1 |
| **History of Tobacco Use** |  |  |  |
| Yes | 1.34 (0.85, 2.12) | 1.12 (0.67, 1.87) | 1.10 (0.61, 1.98) |
| No | 1 | 1 | 1 |
| **History of Substance Use** |  |  |  |
| Yes | 2.78 (1.57, 4.93) | 2.71 (1.48, 4.99) | 0.58 (0.28, 1.22) |
| No | 1 | 1 | 1 |
| **Homelessness** |  |  |  |
| Yes | 3.64 (1.62, 8.21) | 3.86 (1.66, 9.01) | 0.65 (0.25, 1.68) |
| No | 1 | 1 | 1 |
| **History of Incarceration** |  |  |  |
| Yes | 1.12 (0.48, 2.62) | 1.21 (.051, 2.87) | 0.46 (0.12, 1.71) |
| No | 1 | 1 | 1 |
| **Medical History** |  |  |  |
| Chronic Disease | 7.84 (3.11, 19.75) | 6.26 (2.46, 15.91) | 3.37 (0.74, 15.29) |
| * Group A includes all HUPs |  |  |  |
| ** Group B removes deceased HUPs | |  |  |

|  | **HUP Status Group A^*^** | **HUP Status Group B^**^** | **Mortality** |
| --- | --- | --- | --- |
|  | OR (95% CI) | OR (95% CI) | OR (95% CI) |
| **HUP Status** | N/A | N/A | 2.33 (1.11, 4.89) |
| **Age (Years)** | N/A | N/A | 1.05 (1.02, 1.08) |
| **Sex** | N/A | N/A |  |
| Male |  |  | 1.01 (0.55, 1.90) |
| Female |  |  | 1 |
| **Race** |  |  |  |
| Non-Black | 1 | 1 | 1 |
| Black | 1.34 (0.62, 2.87) | 0.48 (0.2, 1.17) | 0.55 (0.24, 1.26) |
| **Community Income Level (dollars)** |  |  |  |
| 0 - 27,651 | 1.01 (0.48, 2.15) | 0.94 (0.44, 2.23) | 1.22 (0.48, 2.97) |
| 27,652 - 39,421 | 0.78 (0.36, 1.70) | 0.83 (0.36, 1.89) | 1.64 (0.66, 4.05) |
| 39, 422 - 48,093 | 0.73 (0.32, 1.65) | 0.62 (0.25, 1.52) | 1.90 (0.76, 4.73) |
| 48, 094 - 139,543 | 1 | 1 | 1 |
| **Payer** |  |  |  |
| Medicaid | 5.22 (1.90, 14.31) | 5.25 (1.80, 15.29) | 2.06 (0.67, 6.36) |
| Medicare | 5.39 (1.96, 14.85) | 4.51 (1.54, 13.17) | 1.48 (0.50, 4.43) |
| Medicare and Medicaid | 1 | 1 | 1 |
| Private | 1.25 (0.30, 5.18) | 0.93 (0.19, 4.63) | 1.40 (0.32, 6.15) |
| Self/None | 4.00 (1.46, 10.96) | 3.59 (1.24, 10.43) | 2.20 (0.68, 7.11) |
| **History of Alcohol Use** |  |  |  |
| Yes | 0.68 (0.37, 1.27) | 0.73 (0.37, 1.44) | 2.20 (1.04, 4.27) |
| No | 1 | 1 |  |
| **History of Tobacco Use** |  |  |  |
| Yes | 1.83 (1.00, 3.35) | 1.64 (0.83, 3.22) | 0.92 (0.49, 1.73) |
| No | 1 | 1 |  |
| **History of Substance Use** |  |  |  |
| Yes | 2.64 (1.27, 5.50) | 2.52 (1.17, 5.44) | 0.68 (0.30, 1.51) |
| No | 1 | 1 |  |
| **Homelessness** |  |  |  |
| Yes | 2.91 (1.12, 7.53) | 2.77 (1.04, 7.37) | 0.84 (0.31, 2.28) |
| No | 1 |  |  |
| **History of Incarceration** |  |  |  |
| Yes | 0.98 (0.34, 2.83) | 0.98 (0.33, 2.86) | 0.34 (0.09, 1.33) |
| No | 1 |  |  |
| **Medical History** |  |  |  |
| Neurological | 2.68 (1.51, 4.79) | 2.29 (1.49, 5.23) | 0.81 (0.44, 1.49) |
| Cardiac | 1.20 (0.59, 2.42) | 1.41 (0.65, 3.08) | 0.94 (0.41, 2.17) |
| Pulmonary | 3.33 (1.87, 5.94) | 3.41 (1.81, 6.40) | 1.54 (0.84, 2.85) |
| GI | 3.21 (1.76, 5.47) | 2.91 (1.51, 5.60) | 1.01 (0.55, 1.85) |
| Hematological | 5.95 (3.25, 10.90) | 4.41 (2.27, 8.59) | 2.22 (1.16, 4.27) |
| Infectious Disease | 2.86 (1.40, 5.86) | 3.81 (1.44, 7.02) | 1.35 (0.65, 2.79) |
| Renal | 3.42 (1.77, 6.64) | 3.66 (1.73, 7.74) | 1.03 (0.54, 1.96) |
| Gynecology-Urology | 1.18 (0.59, 2.37) | 0.95 (0.43, 2.08) | 1.50 (0.77, 2.91) |
| Muscular-Skeletal | 0.76 (0.41, 1.43) | 0.73 (0.37, 1.43) | 0.61 (0.30, 1.22) |
| Psychiatric | 1.51 (0.80, 2.86) | 1.58 (0.80, 3.12) | 0.59 (0.29, 1.22) |
| Endocrine | 1.93 (1.08, 3.46) | 1.97 (1.04, 3.73) | 1.75 (0.95, 3.20) |
| Rheumatology/Nutritional/Other | 0.50 (0.22, 1.11) | 0.56 (0.23, 1.35) | 1.10 (0.48, 2.53) |
| Trauma | 0.76 (0.22, 2.60) | 1.03 (0.29, 3.67) | 0.52 (0.06, 4.38) |
| BMI | 1.01 (0.97, 1.05) | 1.00 (0.96, 1.05) | 0.95 (0.92, 1.00) |

Table S2. Regression analysis demonstrating demographic, social, and all clinical diagnoses associated with high utilizer patient status and mortality

***** Group A includes all HUPs

** Group B removes deceased HUPs
